# Association between post-adenoidectomy hypernasality and velopharyngeal valve closure patterns

**DOI:** 10.1007/s00405-025-09748-4

**Published:** 2025-11-01

**Authors:** Dalia G. Yasien, Rawan K. Alariney, Mohamed ElSheikh, Mohamed G. Hamed

**Affiliations:** 1 Phoniatrics Unit, Department of Otorhinolaryngology, Faculty of Medicine, Helwan University, Cairo, Egypt; 2 Department of Otorhinolaryngology, Helwan University Faculty of Medicine, Cairo, Egypt

**Keywords:** Adenoid, Adenoidectomy, Hypernasality, Resonance, VPV function

## Abstract

**Purpose:**

Hypernasality is a speech disorder that is frequently associated with various pathological conditions. However, its precise prevalence following adenoidectomy remains unclear. This study aimed to investigate the possible relation between velopharyngeal valve (VPV) closure patterns and the development of hypernasality following adenoidectomy.

**Methods:**

A prospective observational study was conducted on 100 children, aged 6–12 years, with adenoid hypertrophy who underwent total adenoidectomy after nasoendoscopic evaluation of velopharyngeal valve function. All participants were evaluated for postoperative hypernasality at 2 weeks and monthly up to 3 months.

**Results:**

Preoperatively, all patients had good intelligibility of speech, 85% of cases had normal resonance, while 15% had a mild degree of hyponasality. Nasofibroscopic examination showed incomplete adenoid closure, adequate palatal length and mobility, and complete velopharyngeal valve closure in all subjects. Regarding velopharyngeal valve closure pattern, 65% of patients exhibited a coronal pattern and 35% a circular pattern. Postoperatively, only 5% of patients developed mild transient hypernasality at the two-week follow-up, while 95% maintained normal resonance. By the one-month assessment, all patients had achieved normal resonance.

**Conclusions:**

There was no significant association between VPV closure patterns and the development of postoperative hypernasality. Meticulous preoperative assessment of VPV function can effectively minimize the risk of post-adenoidectomy hypernasality.

## Introduction

Adenoid hypertrophy is known to compromise the patency of the nasopharyngeal and restrict airflow [1]. This anatomical obstruction can contribute to various complications, including nasal obstruction, sleep-disordered breathing, and recurrent otitis media. Adenoidectomy, either alone or with tonsillectomy, is a common procedure in pediatric otorhinolaryngology [2]. 

Velopharyngeal dysfunction (VPD) refers to inadequate velopharyngeal closure during speech or swallowing, leading to symptoms such as hypernasality, audible nasal air emission, and nasal regurgitation of food or fluids during swallowing. VPD is classified into: (1) velopharyngeal insufficiency (VPI) resulting from structural abnormalities, (2) velopharyngeal incompetence, due to neuromuscular dysfunction, and (3) velopharyngeal mislearning, where there is inappropriate articulatory placement [3].

During speech, the soft palate typically elevates against the adenoid, preventing air from escaping into the nasal cavity. Adenoidectomy may enlarge the velopharyngeal port, potentially disrupting this mechanism and resulting in hypernasality [4].

Hypernasality, often associated with various pathological conditions, can negatively affect communication and psychosocial well-being for both children and their families [5].

While persistent hypernasality is rare and usually linked to underlying anatomical anomalies, transient hypernasality is a relatively common postoperative complication following adenoidectomy. However, its exact incidence remains uncertain [6].

Flexible fiberoptic nasoendoscopy is an effective tool for evaluating the dynamics of the velopharyngeal mechanism. It allows for direct visualization of adenoid size, VPV closure grade, and pattern during speech [7].

This study aimed to determine the possible relation between velopharyngeal valve (VPV) closure patterns and the development of hypernasality following adenoidectomy.

## Patients and methods

This prospective observational study was conducted on 100 children aged 6 to 12 years old, of both sexes, all diagnosed with adenoid hypertrophy associated with either recurrent otitis media with effusion or sleep-disordered breathing. The study was conducted following the ethical principles outlined in the Declaration of Helsinki of 1964. This study was conducted between March 2024 and November 2024, in Helwan University Hospital (Badr Hospital). Ethical approval was obtained from the research ethics committee of the Faculty of Medicine- Helwan University on 5/2/2024, Serial no: 25–2024. Written informed consent was obtained from the parents of all participants prior to their inclusion in the study.

Exclusion criteria included children younger than 6 or older than 12 years, children with craniofacial anomalies, palatal abnormalities, preoperative hypernasality or mixed nasality (open nasality with closed nasality), and patients with incomplete VPV closure confirmed by nasofibroscopy. Children younger than 6 years were excluded due to expected difficulties in cooperation during nasofibroscopic assessment. Children older than 12 years were excluded to avoid the potential influence of post-pubertal anatomical and functional changes that may affect velopharyngeal function [8].

Preoperatively, all patients underwent comprehensive evaluations including medical history, general and ENT examination, routine laboratory investigations, auditory perceptual assessment of speech (APA), and nasopharyngoscopic evaluation of VPV function.

Auditory perceptual assessments (APA) of speech were performed in Phoniatrics unit to detect abnormal resonance, determine the type of nasality (Open, closed, or mixed), degree of nasality; (0 = normal, 1 = mild, 2 = moderate, 3 = severe), and the degree of speech intelligibility; (0 = good, 1 = mild, 2 = moderate, 3 = severe). Simple clinical tests were also conducted, including Gutzmann’s (a/e) test and Czermak’s (Cold mirror) test [9].

Nasopharyngoscopic assessments by FENTEX 700,710 FX- Germany flexible nasofibroscope were performed in the Phoniatrics unit to evaluate VPV closure function and determine VPV closure pattern (coronal, circular, or sagittal).

All participants underwent total adenoidectomy using the conventional curettage technique, either as adenoidectomy alone or in combination with tonsillectomy. Postoperative APA of speech was conducted in the Phoniatrics unit at 2 weeks, 1 month, 2 months, and 3 months following surgery.

### Statistical analysis

Data analysis was conducted using IBM SPSS Statistics version. Quantitative variables were expressed as mean ± standard deviation (SD), range, and median with inter-quartile range (IQR), and were analyzed using the Friedman test for repeated measures. Categorical variables were reported as frequencies and percentages, and comparisons were made using the Wilcoxon signed-rank test. A *P*-value of < 0.05 was considered statistically significant.

## Results

The ages of the study participants ranged from 6 to 12 years, with a mean of 8.68 ± 1.59 years. Regarding sex distribution, 61% were male and 39% were female. As for tonsillar status, 90% had hypertrophied tonsils, while 10% had a history of tonsillectomy. Consequently, 85% underwent adenotonsillectomy and 15% had adenoidectomy alone. Of those with hypertrophied tonsils, 5% did not meet the surgical criteria of tonsillectomy.

In the preoperative APA, normal resonance was identified in 85% of cases, whereas mild hyponasality was observed in the remaining 15%. All participants presented with good speech intelligibility. Gutzmann’s (a/e) and Czermak’s (Cold mirror) tests were negative [Table 1]. 

**Table 1:** Pre-operative auditory perceptual assessment of speech (*n* = 100)

Variable		*N*	%
Resonance:	Normal Hyponasality Hypernasality Mixed nasality	85 15 0 0	85.0% 15.0% 0.0% 0.0%
Degree of Hyponasality:	Absent Mild Moderate Severe	85 15 0 0	85.0% 15.0% 0.0% 0.0%
Intelligibility of speech	Good Mild unintelligibility Moderate unintelligibility Severe unintelligibility	100 0 0 0	100.0% 0.0% 0.0% 0.0%

Preoperative nasofibroscopic examination showed incomplete adenoid closure (partial choanal obstruction), adequate palatal length and mobility, and complete VPV closure in all cases. Regarding VPV closure pattern, 65% of patients had a coronal closure pattern and 35% had a circular closure pattern [Table 2].

**Table 2:** Preoperative nasofibroscopic assessment (*n* = 100)

Variable		*N*	%
Adenoid closure	Incomplete Complete	100 0	100.0**%** 0.0**%**
Palatal mobility	Good Mild impairment Moderate impairment Severe impairment	100 0 0 0	100.0**%** 0.0% 0.0% 0.0%
VPV closure	Complete Incomplete	100 0	100.0% 0.0%
VPV closure pattern	Coronal Circular Sagittal	65 35 0	65.0% 35.0% 0.0%

Postoperatively, 5% of patients developed mild transient hypernasality at the two-week follow-up, while the remaining 95% maintained normal resonance. Among the 5% of patients who developed mild transient hypernasality at the two-week postoperative follow-up, all had undergone adenotonsillectomy. Of these five patients, three had a coronal VPV closure pattern, while the remaining two had a circular closure pattern. By the one-month assessment, all patients had achieved normal speech resonance, which was maintained at the two- and three-month follow-ups. The improvement in APA over time was statistically significant (Friedman test, *p* = 0.002) [Table 3].

**Table 3:** Post-operative auditory perceptual assessment (APA) over time (*n* = 100)

Variable	Postoperative follow-up							
	2 weeks		1 month		2 months		3 months	
	*N*	%	*N*	%	*N*	%	*N*	%
Normal resonance	95	95.0%	100	100.0%	100	100.0%	100	100.0%
Hyponasality	0	0.0%	0	0.0%	0	0.0%	0	0.0%
Mild hypernasality	5	5.0%	0	0.0%	0	0.0%	0	0.0%
Moderate hypernasality	0	0.0%	0	0.0%	0	0.0%	0	0.0%
Severe hypernasality	0	0.0%	0	0.0%	0	0.0%	0	0.0%
Fr	15							
P	0.002*							

Friedman test: Fr = 15. *P*- value = 0.002*, Statistically significant at *P* < 0.05

## Discussion

The relationship between velopharyngeal closure function and hypernasality following adenoidectomy has been a subject of increasing interest. Variations in closure dynamics, such as incomplete closure or inefficient movement, may increase the risk of postoperative hypernasality. Understanding these anatomical and functional variations helps clinicians to predict potential speech complications and plan appropriate management [10].

Although Children with normal VPV function are not supposed to develop velopharyngeal insufficiency and consequently hypernasality, temporary postoperative alterations in VPV functions may occur, despite the absence of any anatomical or neuromuscular deficits [11]. As a result, transient hypernasality is frequently observed following adenoidectomy; however, there is limited knowledge regarding the incidence of this alteration and how long it lasts [6]. Some researchers have documented that it can last for several months [12–14].

Moreover, there is a lack of research exploring whether the preoperative VPV closure pattern might influence postoperative resonance outcomes.

Our findings did not demonstrate a relationship between velopharyngeal valve closure patterns and the development of post-adenoidectomy hypernasality. Only 5% of cases (three with coronal and two with circular patterns) exhibited mild hypernasality, which resolved within one month postoperatively, suggesting that the condition was not related to the underlying VPV closure pattern. Similarly, a recent study by Elwany et al. concluded that closure patterns had no significant value in predicting postoperative hypernasality [15].

Regarding the most reported VPV closure pattern, our findings revealed that the coronal pattern (65%) was more prevalent than the circular pattern (35%), with no sagittal closure pattern among our participants. Similarly, Elwany et al. reported a slightly higher prevalence of the coronal closure (52%), compared to the circular pattern (43%), and sagittal in only 3% [15]. These findings differ from those reported by El-Anwar et al., who found nearly equal distribution between coronal (50.5%) and circular (49.5%), with no sagittal or circular with Passavant’s ridge patterns [16]. In contrast, Manochiopinig et al. identified the circular closure as the most common pattern in their study [17]. These variations across studies may be attributed to differences in age distribution and possibly regional differences.

Regarding postoperative auditory perceptual assessment, the mild degree of hypernasality observed in 5% of cases at the two-week follow-up could be attributed to postoperative pain and edema, which may have temporarily affected palatal mobility and VPV closure. By the one-month evaluation, all of these patients had regained normal resonance.

Our findings align with those of Abdel-Aziz et al., who reported full recovery of normal resonance in all patients by the third postoperative month, except for one case (1.4%) that required speech therapy and extended follow-up due to persistent hypernasality [10].

This means that when all risk factors are excluded by detailed velopharyngeal valve assessment before adenoidectomy, the possibility of hypernasality becomes minimal and transient. Additionally, the identification of high-risk factors, such as submucous cleft, short palate, and deep pharynx, may play a crucial role in guiding the surgeon’s decision-making process. In such cases, a partial adenoidectomy, rather than a total adenoidectomy, may be considered. This involves removal of only the upper part of the adenoid tissue obstructing the choana, while preserving the lower portion to support VPV closure [18]. Preserving a portion of the adenoid tissue could play a crucial role in maintaining velopharyngeal competence, thereby preventing the development of hypernasality following surgery in high-risk subjects. This is supported by the findings of Gelaney et al., who reported the absence of postoperative hypernasality following partial adenoidectomy across all high-risk subjects of their study [19].

Based on **our** findings, we recommend conducting future studies on a larger sample size to further explore the risk factors associated with post-adenoidectomy velopharyngeal insufficiency (VPI). Through preoperative assessment of adenoid size and percentage of its nasopharyngeal closure, palatal length and mobility, and VPV closure grades and patterns is strongly advised. Such an assessment is critical to identifying patients at higher risk of developing hypernasality and guiding surgical decision-making.

## Conclusions

No significant association was found between VPV closure patterns and the development of hypernasality following adenoidectomy.

Transient post-adenoidectomy hypernasality occurred in 5% of cases and resolved within one month. These findings suggest that thorough preoperative assessment can help minimize the risk of postoperative hypernasality.

## Limitations

Auditory perceptual assessments and nasopharyngoscopic evaluations were done by a single observer. In addition, the study did not elaborate on the impact of adenoid size and the age of participants on the development of postoperative hypernasality.

## Data Availability

The data that support the findings of this study are available on request from the corresponding author.
